# Unilateral upper limb chorea associated with hyperthyroidism: A case report and literature review

**DOI:** 10.3389/fneur.2022.1074156

**Published:** 2022-12-22

**Authors:** Wei Chen, Bin Wu, Hongna An, Kaiying Zheng, Daming Zhai, Jiahua Zang, Xiaobing Wu

**Affiliations:** Department of Neurology, The Second People's Hospital of Quzhou, Quzhou, China

**Keywords:** chorea, involuntary movement, hyperthyroidism, Graves' disease, initial presentation

## Abstract

Chorea, a hyperkinetic syndrome, is generally reported in patients with Huntington's disease (HD), hyperglycemia, and other diseases but occasionally occurs in patients with Grave's disease. Here, we report a 44-year-old woman presenting with a 1-year history of involuntary movements with a known history of primary hyperthyroidism. Physical examination revealed the continuous, rapid, irregular, and spontaneous choreic movement of her right arm. Laboratory investigations demonstrated increased triiodothyronine (T3) and free thyroxine (FT4) and suppressed thyroid-stimulating hormone (TSH) levels. An electroencephalogram and brain magnetic resonance imaging were normal. After antithyroid treatment, the patient achieved complete remission. Our case indicated that hemichorea might initially manifest hyperthyroidism. Therefore, thyroid function tests should be routinely performed in patients with chorea.

## Introduction

Chorea is a hyperkinetic syndrome characterized by irregular, brief and non-stereotyped movements resulting from abrupt twitching of the muscles, which flit from one body region to another (1). Chorea is commonly described in patients with Huntington's disease (HD) (2), hyperglycemia (3), autoimmune thyroid disease, drug toxication (4), etc. (5). However, chorea is a rare complication of hyperthyroidism, with <2% of chorea cases occurring in patients with Grave's disease (6). In this report, we present a case of hemichorea as the first manifestation of hyperthyroidism which was resolved with antithyroid therapy.

The study was performed according to the principles of the Helsinki declaration and the local ethical standards. Written informed consent was obtained from the patient.

## Case report

A 44-year-old female patient was admitted to our neurology department in October 2021 due to a one-year history of involuntary movements of her right arm. She had been diagnosed with uncontrolled hyperthyroidism due to Grave's disease in April 2021 and treated with methimazole (10 mg daily). There was an obvious improvement in her involuntary movements 1 month later, but she had to stop taking methimazole due to the development of urticarial and liver dysfunction. She has not received other antithyroid drugs or radioiodine treatment for hyperthyroidism.

After the discontinuation of methimazole, her involuntary movements gradually deteriorated, with symptoms occurring at any time, even during sleep. In addition, she complained of mild palpitations, irritability, and anxiety. By the time of admission, the choreiform movements were continuous, rapid, irregular, and spontaneous (Supplementary Video S1), and her right arm was totally out of control. She had lost weight and had a rapid heart rate of 126 beats per minute. Neurological examination was otherwise normal except for choreic movements predominating in the right upper limb.

Laboratory results revealed increased triiodothyronine (T3) level (8.26 nmol/L, normal range: 0.92–2.79 nmol/L) and free thyroxine (FT4) level (58.13 pmol/L, normal range: 11.50–22.70 pmol/L), suppressed thyroid-stimulating hormone (TSH) level (<0.01 mIU/L, normal range: 0.55–4.78 mIU/L), and a positive anti-thyroglobulin antibody titer (105.20 KIU/L, normal range: 0–60.00 KIU/L). Hematologic investigations, including a complete blood cell count, liver and kidney functions, glucose level, autoantibody titers, serum tumor markers, ceruloplasmin, and creatine kinase activity, were normal. Thyroid ultrasonography showed diffuse heterogeneity, focal hypoechogenicity of the thyroid gland, and a diffusely enhanced thyroid blood flow. The electroencephalogram was within normal limits. Brain magnetic resonance imaging (MRI) showed normal axial T1, T2, and DWI. Lumbar puncture confirmed normal opening pressure, and no abnormality was observed in the cerebrospinal fluid analysis.

Considering her methimazole intolerance, she was treated with radiation (^131^I) therapy. Her symptoms gradually resolved (Supplementary Video S2), along with slightly decreased T3 and T4 and elevated TSH levels. The chorea movement almost disappeared 3 months later, and there was no recurrence after 6 months of follow-up.

## Literature review and discussion

This unusual case of hemichorea secondary to hyperthyroidism was resolved with a (^131^I) regimen. Chorea is an abnormal movement disorder typically manifesting as continual involuntary, abrupt, rapid, brief, and irregular movements that randomly flow from one body part to another in a non-stereotyped mode. In rare instances, chorea is related to poorly controlled hyperthyroidism, which was first reported by Gowers in 1983 (7).

We reviewed case reports of hyperthyroid-related chorea published between January 1990 and August 2022, identifying 27 cases of chorea due to hyperthyroidism. The clinical characteristics of all 28 cases, including our case, are presented in Table 1. The median age of patients with hyperthyroid chorea was 23 years old (range, 8–78 years) and was reported in 22 females (77.8%) and six males, five from China, four from the United States, three from Japan, and three from South Korea. Hyperthyroid-related chorea is typically manifested by acute or subacute and progressive choreiform movements with predominant distal involvement. The involuntary movements symmetrically (17 cases) or asymmetrically involve arms and legs, predominating on the left side (11 cases), and are more pronounced in the leg while walking, causing infrequent falls. The trunk, face, and bucco-oral-lingual region can also be affected, resulting in speech disturbance and dysphagia. Thyrotoxic symptoms, including weight loss, palpitations, sweating, and anxiety, usually appear weeks to years before involuntary movements. Neurological examinations are commonly normal, but some cases reported brisk deep tendon reflexes. The characteristics of hyperthyroid chorea on neuroimaging, including CT, MRI, and MRA of the brain, were normal, and the brain MRI and MRA of the current case also revealed no structural changes. In our case, the patient presented with acute onset of worsening involuntary movements of her right arm.

**Table 1:** Clinical characteristics of 28 patients with hyperthyroidism-associated chorea.

**Age/sex (ref)**	**Publication year/country**	**Clinical presentation**	**Medical history**	**Neurological syndromes**	**Disease duration**	**Treatment**	**Prognosis**
44/F (PR)	2022/China	Involuntary movements of her right arm	Grave's disease	Normal	1 year	Radiation (^131^I) therapy	Improvement within 3 months
67/M (8)	2022/Canada	Ongoing, non-distractible choreiform movements of the left upper extremity	Right frontoparietal stroke, thyroidectomy with ablation, thyroid hormone replacement	Spasticity, mildly reduced strength, and 3+ hyperreflexia/left	NR	L-thyroxine (dosage was decreased to 20 mg twice a day), beta blocker	Improvement within 2 months
14/M (9)	2021/Kenya	Chorea, tremors, a low BMI of 17	Unremarkable	Tremor/bilateral limbs	A few months	Gabapentin, carbimazole, and radioactive iodine therapy	Improvement
13/F (10)	2020/USA	Worsening left-sided upper extremity weakness and gait unsteadiness for 1 month	Unremarkable	Tremors, gait alterations with left foot drop, slurred speech/left	1 month	Methimazole and propranolol	Improvement within 12 months
8/F(10)	2020/Argentina	Subacute onset lost weight, gained height, involuntary movements	Asthma	Lingual fasciculations, dysarthria/bilateral limbs	1 month	Methimazole, atenolol and carbamazepine	Improvement within 1 month
62/M (11)	2019/China	Asymmetric involuntary movement, muscle weakness, inaccurate coordinate movement, and hypomyotonia of right limbs	Diabetes, atrial fibrillation	Normal	2 weeks	Methimazole, haloperidol, and bisoprolol	Improvement within 2 weeks
60/F (12)	2019/Switzerland	Erratic, intricate movement disorders in her left upper and lower extremities	Hypertension	Tremor/left	5 years	Carbimazole and propylthiouracil (switched to radioiodine therapy due to severe adverse effects)	Improvement within 2 months
32/F (13)	2016/India	Jerky, non-repetitive involuntary movements of the left upper and lower limbs	Unremarkable	Normal	NR	Carbimazole	Improvement within 6 weeks
25/F (14)	2016/USA	Muscle spasms of the left shoulder and arm	Hyperthyroidism, anxiety, bipolar disorder, depression, substance abuse	Normal	NR	Metoprolol and methimazole	Improvement within 1 week
60/F (15)	2015/Switzerland	Imbalance associated with falls evolving for 5 years	Unremarkable	Normal	5 years	Carbimazole, ^131^I radiotherapy	Improvement within a few weeks
15/F (16)	2013/Canada	A series of falls over the month, choreiform episodes, insomnia, fatigue, and loss of appetite	Sickle cell disease	Normal	2 weeks	Methimazole first, then methimazole plus levothyroxine	Improvement
23/F (17)	2013/Poland	Palpitations, weight loss, and exercise intolerance	Unremarkable	Normal	NP	Thiamazole, Haloperidol, Prednisone	Improvement within 6 weeks
22/F (18)	2013/South Korea	Involuntary movement of her four extremities	Hyperthyroidism	Dysarthria	2 months	Propylthiouracil	Improvement within 1 month
16/M (19)	2012/South Korea	Choreic movement dominant in the right limb	Unremarkable	Brisk deep tendon reflex/bilateral limbs	9 days	Propylthiouracil, propranolol	Improvement within 8 months
14/M (20)	2012/China	Acute onset, generalized proximal muscle weakness, and hyporeflexia	Unremarkable	Normal	5 hours	Methimazole	Improvement within 4 weeks
23/F (21)	2011/USA	Involuntary, writhing, symmetrical movements involving arms, legs, neck, tongue, and face starting 10 days following delivery of her second child. weight loss	Toxic nodular goiter	Normal	NR	Propylthiouracil, atenolol, quetiapine, and a short prednisone taper	Improvement within 6 months
38/F (22)	2010/China	Ilateral blepharospasm with visual difficulty, and facial grimacing. involuntary choreic movements in her left side	Unremarkable	Bilateral blepharospasm, oromandibular dystonia; Irregular speech in volume and tempo, irregular and unsteady gait	3 months	Methimazole	Improvement within 4 months
17/F (23)	2009/China	Acute onset, involuntary movement of hands, forearms, feet, and face for 2 weeks	Graves' disease	Irregular and unsteady gait, irregular speech in volume and tempo	2 weeks	Propylthiouracil, propranolol, and haloperidol	Improvement within 6 weeks
42/F (24)	2008/South Korea	Continuous, involuntary movement in her left upper extremity and face for 1 month	Graves' disease	Normal	1 month	Methylprednisolone sodium succinate and oral antithyroid medication	Improvement
19/F (25)	2008/France	About 2 weeks before admission, she had progressively developed movement disorder, balance impairment, and dysarthria	Graves' disease	Mild dysarthria and impaired tandem walk	2 weeks	Carbimazole and levothyroxine	Improvement within 3 months
9/F (26)	2007/USA	A 2 month-history of weight loss, hyperactivity, tremulousness, and palpitations	Unremarkable	Ataxic gait, dysmetria, and dysdiadochokinesia	2 months	Propylthiouracil and propranolol	Improvement within 4 days
78/F (27)	2005/UK	A 1-week history of increasing agitation and worsening generalized involuntary movements	IHD, AF, AS, hypercholesterolemia, and total thyroidectomy	Impaired speech and dysphagia	1 week	Propranolol; discontinuation of thyroxine	Improvement within 3 months
50/M (6)	2004/Yugoslavia	Sudden development of vigorous bilateral, ballistic, and severe choreic movements of all limbs, more prominent on the left side	Hyperthyroidism	Normal	NR	Haloperidol, propranolol and thiamazole	Improvement within 10 days
23/F (28)	2003/Japan	Marked sweating, irritability, poor concentration, and tremors in both hands	Parkinson's disease	Right-hand tremor	15 months	Methimazole and β-adrenoceptor blocker	Improvement within 2 weeks
24/F (29)	1998/Japan	Acute left-sided chorea and dysarthria	Graves' disease	Normal	NR	Methimazole, propranolol, and diazepam	Improvement within 4 weeks
Elderly female (30)	1994/Italy	Chorea	Hyperthyroidism	Normal	NR	-	Improvement
16/F (31)	1992/Italy	Depression of the mood, tremors, motor incoordination, and chorea more evident in the right side	Operated on for closure of a ventricular septal defect, Graves' disease	Muscular hypotonia, decreased tendon reflexes, facial grimacing, and dysarthria/right	8 months	Methimazole	Improvement within 2 months
23/F (32)	1992/Japan	Severe involuntary movements in the left extremities	Unremarkable	Normal	2 years	Thiamazole and propranolol	Improvement within 2 months

F, female; M, male; PR, present case; NR, not reported; IHD, ischemic heart disease; AF, atrial fibrillation; AS, aortic stenosis.

To date, the physiopathologic mechanisms of hyperthyroid-related chorea remain elusive. It has been suggested that chorea may result from a direct effect of thyrotoxicosis on the central nervous system in Graves' disease. Structural changes in the basal ganglia have not been demonstrated postmortem (33), which is consistent with the normal neuroimaging of the previously reported cases. Hypersensitivity of the dopaminergic system in the nigrostriatal pathway of basal ganglia has been suggested to be one of the underlying mechanisms. Homo-vanillic acid, a dopamine metabolite, was significantly decreased in the cerebrospinal fluid of hyperthyroid patients (34). Moreover, treatment with dopamine antagonists can alleviate the symptoms of hyperthyroidism-related chorea (35). Functional modification of adrenergic receptors may also be involved in hyperthyroid-related chorea (14, 28), which is also supported by the partially relieved chorea with propranolol (a non-selective β1 and β2 adrenergic receptor blocker) treatment. ^18^F-fluorodeoxyglucose positron emission tomography (FDG-PET) showed elevated metabolism in the bilateral basal ganglia in a patient whose choreic movements predominately involved her right side (18), suggesting that hyperthyroidism may have a direct thyrotoxicosis effect resulting in excessive dopaminergic activity in the basal ganglia.

Treating hyperthyroidism-associated chorea consists of correcting thyroid function with antithyroid drugs and adding symptomatic agents, if necessary. In most patients, the choreic movements gradually improved over weeks or months with the normalization of their thyroid function. Although some patients must stop the antithyroid drug because of adverse effects such as severe muscle pain and myalgia, significant clinical alleviation was noticed in parallel with their improved thyroid function. These patients also benefited from radioiodine or thyroidectomy. Hence, it is indicated that hyperthyroidism-associated chorea is reversible after treatment with beta-adrenergic blockers, dopamine antagonists, and especially antithyroid drugs.

## Conclusion

In summary, hemichorea is rare in hyperthyroidism patients and may be the initial manifestation of hyperthyroidism. Therefore, it is recommended that thyroid function tests should be routinely performed in patients with chorea. The rapid resolution of the chorea after controlling the hyperthyroidism in the absence of any structural lesion suggests that the movement disorder was likely a result of thyrotoxicosis-induced biochemical changes rather than the coexistence of a structural lesion. Further studies are needed to explore the etiology and pathogenesis of hyperthyroidism-induced chorea.

## Data availability statement

The original contributions presented in the study are included in the article/Supplementary material, further inquiries can be directed to the corresponding author.

## Ethics statement

The studies involving human participants were reviewed and approved by the Ethical Committee of the Second People's Hospital of Quzhou. The patients/participants provided their written informed consent to participate in this study. Written informed consent was obtained from the individual(s) for the publication of any potentially identifiable images or data included in this article.

## Author contributions

WC designed the work and wrote the original draft. WC, BW, HA, KZ, DZ, JZ, and XW initiated the project, collected, and analyzed the data. XW wrote the review, edited, supervised, and validated the manuscript. All authors read and approved the final manuscript.

## References

[1] Burn D. Oxford Textbook of Movement Disorders. London: BMJ Publishing Group Ltd. (2015).

[2] Talukder P Jana A Dhar S Ghosh S. Huntington's chorea-a rare neurodegenerative autosomal dominant disease: insight into molecular genetics, prognosis and diagnosis. Appl Biochem Biotechnol. (2021) 193:2634–48. 10.1007/s12010-021-03523-x 34235640

[3] Sasaki T Suzuki Y Sato M. Hyperglycemic chorea. Oxf Med Case Rep. (2021) 2021:omab118. 10.1093/omcr/omab118 34909206 PMC8665685

[4] Laban T Larroche C Comparon C Dhote R Degos B. Fluoxetine-induced chorea. Rev Neurol. (2021) 177:1010–1. 10.1016/j.neurol.2020.12.007 34148736

[5] Bovenzi R Conti M Cerroni R Pierantozzi M Stefani A Pisani A. Adult-onset sporadic chorea: real-world data from a single-centre retrospective study. Neurol Sci. (2021) 43:387–92. 10.1007/s10072-021-05332-w 34041635 PMC8724109

[6] Ristić AJ Svetel M Dragasević N Zarković M Koprivsek K Kostić VS. Bilateral chorea-ballism associated with hyperthyroidism. Mov Disord. (2004) 19:982–3. 10.1002/mds.20119 15300671

[7] WR G. A manual of the diseases of the nervous system. JAMA. (1893) xxi:950. 10.1001/jama.1893.0242077004001325996397

[8] Lun R Moores M Mestre T Breiner A. Thyrotoxicosis resulting in unilateral upper limb chorea and ballismus. Can J Neurol Sci. (2022) 49:431–2. 10.1017/cjn.2021.136 34134800

[9] Mongare N Njenga E Sokhi D. Medically refractory Graves' disease presenting as chorea and carbohydrate hyperphagia in a Kenyan adolescent. Mov Disord. (2021) 36(SUPPL 1):S25.

[10] Vundamati D Tsai S. Simultaneous onset of autoimmune hyperthyroidism and rheumatic fever in an adolescent female-case report. US Endocrinol. (2020) 16:125–8. 10.17925/USE.2020.16.2.125

[11] Li X Wang R He J Xu L. Unilateral chorea secondary to hyperthyroidism: a case report. Int J Clin Exp Med. (2019) 12:7870–3.

[12] Delhasse S Debove I Arnold-Kunz G Ghika JA Chabwine JN. Erratic movement disorders disclosing Graves' disease and paralleling thyroid function but not autoantibody levels. J Int Med Res. (2019) 47:1378–86. 10.1177/0300060518816873 30761931 PMC6421382

[13] Raghavendra Rao S Seshadri S Karthik Rao N Patil N Kunder SK Avinash A. Hemichorea: an unusual manifestation of thyrotoxicosis. Asian J Pharm Clin Res. (2016) 9.

[14] Arifi B Gupta S Sharma S Daraboina A Ahuja S. A case report of chorea associated with hyperthyroidism. J Clin Diagn Res. (2016) 10:PL01. 10.7860/JCDR/2016/17299.7277 27042533 PMC4800599

[15] Delhasse S Debove I Ghika JA Chabwine JN. Erratic movement disorders disclosing Grave's disease. Schweiz Arch Neurol Psychiatr. (2015) 166(Supplement 7):66.30761931

[16] Leblicq C Duval M Carmant L Van Vliet G Alos N. Rising serum thyroxine levels and chorea in graves' disease. Pediatrics. (2013) 131:e616–9. 10.1542/peds.2012-0686 23296438

[17] Kaminska A Kaminski M Nowaczewska M Ksiazkiewicz B Junik R. Chorea, convulsive seizures and cognitive impairment in a patient with autoimmune thyroid disease. Neurol Sci. (2013) 34:263–4. 10.1007/s10072-012-0990-4 22402791 PMC3562547

[18] Chung EJ Bae SK Kim SJ. Generalized chorea with abnormal 18F-fluorodeoxyglucose positron emission tomography findings in a patient with hyperthyroidism. Clin Neurol Neurosurg. (2013) 115:108–9. 10.1016/j.clineuro.2012.04.031 22831908

[19] Park J Kim JG Park SP Lee HW. Asymmetric chorea as presenting symptom in Graves' disease. Neurol Sci. (2012) 33:343–5. 10.1007/s10072-011-0679-0 21796431

[20] Nakavachara P Suppakrucha M Likasitwattanakul S. Thyrotoxic periodic paralysis and chorea: two uncommon neuromuscular complications in an adolescent with newly diagnosed Graves disease. In: International Journal of Pediatric Endocrinology Conference: 7th Asia Pacific Paediatric Endocrine Society Biennial Scientific Meeting, APPES. (2012). 10.1186/1687-9856-2013-S1-P145

[21] Masannat Y Gandhy R Olajide O Kheetan R Yaqub A. Chorea associated with thyrotoxicosis due to toxic multinodular goiter. Thyroid. (2011) 21:1279–80. 10.1089/thy.2011.0018 21936672

[22] Miao J Liu R Li J Du Y Zhang W Li Z. Meige's syndrome and hemichorea associated with hyperthyroidism. J Neurol Sci. (2010) 288:175–7. 10.1016/j.jns.2009.10.018 19883923

[23] Yu JH Weng YM. Acute chorea as a presentation of Graves disease: case report and review. Am J Emerg Med. (2009) 27:369.e1–3. 10.1016/j.ajem.2008.05.031 19328390

[24] Ku CR Park HJ Hong SJ Shin DY Lee JH Chung MJ. A case of Graves' disease presenting with Chorea. Endocrinol Metab. (2008) 23:342–6. 10.3803/jkes.2008.23.5.34221796431

[25] Garcin B Louissaint T Hosseini H Blanc R Fenelon G. Reversible chorea in association with Graves' disease and moyamoya syndrome. Mov Disord. (2008) 23:620–2. 10.1002/mds.21941 18186119

[26] Seeherunvong T Diamantopoulos S Berkovitz GD. A nine year old girl with thyrotoxicosis, ataxia, and chorea. Brain Dev. (2007) 29:660–1. 10.1016/j.braindev.2007.04.002 17524583

[27] Isaacs JD Rakshi J Baker R Brooks DJ Warrens AN. Chorea associated with thyroxine replacement therapy. Mov Disord. (2005) 20:1656–7. 10.1002/mds.20603 16078207

[28] Hayashi R Hashimoto T Tako K. Efficacy of propranolol in hyperthyroid-induced chorea: a case report. Mov Disord. (2003) 18:1073–6. 10.1002/mds.10477

[29] Nagaoka T Matsushita S Nagai Y Kobayashi K. A woman who trembled, then had chorea. Lancet. (1998) 351:1326. 10.1016/S0140-6736(97)11126-6 9643796

[30] Lucantoni C Grottoli S Moretti A. Chorea due to hyperthyroidism in old age. A case report. Acta Neurol. (1994) 16:129–33. 7992662

[31] Pozzan GB Battistella PA Rigon F Zancan L Casara GL Pellegrino PA. Hyperthyroid-induced chorea in an adolescent girl. Brain Dev. (1992) 14:126–7. 10.1016/S0387-7604(12)80102-1 1621927

[32] Baba M Matsunaga M Terada A Hishida R Takebe K Kawabe Y. Persistent hemichorea associated with thyrotoxicosis. Intern Med. (1992) 31:1144–6. 10.2169/internalmedicine.31.1144 1421727

[33] Waldenström J. Acute thyrotoxic encephalo- or myopathy, its cause and treatment. Acta Med Scand. (1945) 121:251–94. 10.1111/j.0954-6820.1945.tb06882.x

[34] Klawans HL Shenker DM. Observations on the dopaminergic nature of hyperthyroid chorea. J Neural Transm. (1972) 33:73–81. 10.1007/BF01244729 4264577

[35] Baba M Terada A Hishida R Matsunaga M Kawabe Y Takebe K. Persistent hemichorea associated with thyrotoxicosis. Intern Med. (1992) 31:1144–6. 10.2169/internalmedicine.31.1144 1421727

